# Book Notices

**Published:** 1881-12-20

**Authors:** 


### *BOOK NOTICES.*

CYCLOPEDIA OF THE PRACTICE OF MEDICINE.—Edited by Dr. H. Von Ziemssen, Professor of Clinical Medicine in Munich, Bavaria. Vol. XX. New York: Wm. Wood & Co. McGarity & Laird, Agents, Atlanta, Ga.

The above, or twentieth volume, contains the general index of the entire series of this great work. The practitioner who obtains the twenty volumes of this work will have a complete library of the department of practice, including the important advances up to a recent period.

A PRACTICAL TREATISE ON HERNIA, by Joseph H. Warren, M. D., Member American Medical Association, British Medical Association, Massachusetts Medical Society, formerly Surgeon and Medical Director United States Army, etc., etc. Second and revised edition. Fully illustrated. Boston: Jas. A. Osgood & Co. London: Samson Low, Marston, Searle and Rviington, 1882.

An able and elaborate work of 442 octavo pages, embracing the following interesting subjects: 1st. Causation of Hernia—kinds and frequency of Hernia—Anatomy of Hernia—Operations for Hernia, Treatment, etc.—Kelotomy or Herniotomy—Recent operations—Artificial Anus and Wounds of Intestines, Trusses, Hydrocele and Varicocele, Clinical Reports, etc.

WALSHE'S PHYSICIAN'S HANDY LEDGER, Published by Ralph Walshe, M. D., 332 C Street Northwest, Washington, D. C.

"The Handy Ledger is a companion to the Call-Book, and is designed to assist the hurried and busy practitioner in keeping his accounts. The plan of the book is so simple, yet complete, that but a half hour per week is needed to keep a large practice posted up. It is equally well adapted to the wants of city or country practice, and will accommodate six and twelve hundred names."

We will send the above valuable Ledger for 300 patients to any one who will send us the name of a new subscriber enclosing $4.25. R. C. WORD, M. D.

Managing Editor Southern Medical Record.

